# Hepatoprotective Activity of InlB321/15, the HGFR Ligand of Bacterial Origin, in CCI4-Induced Acute Liver Injury Mice

**DOI:** 10.3390/biomedicines7020029

**Published:** 2019-04-11

**Authors:** Yaroslava Chalenko, Konstantin Sobyanin, Elena Sysolyatina, Konstantin Midiber, Egor Kalinin, Alexandra Lavrikova, Lyudmila Mikhaleva, Svetlana Ermolaeva

**Affiliations:** 1 Gamaleya National Research Center of Epidemiology and Microbiology, 123098 Moscow, Russia; yaroslavazaka@yandex.ru (Y.C.); dr.konstsob@yandex.ru (K.S.); demiurg_84@mail.ru (E.S.); kalinin.egor@bk.ru (E.K.); sasha-lavrikova@ya.ru (A.L.); 2 Research Institute of Human Morphology, 117418 Moscow, Russia; midiberkonst@gmail.com (K.M.); mikhalevalm@yandex.ru (L.M.)

**Keywords:** acute toxic liver damage, hepatoprotective effect, HGFR, HGF, bacterial protein

## Abstract

HGF (hepatocyte growth factor)/HGFR (HGF receptor) signaling pathway is a key pathway in liver protection and regeneration after acute toxic damage. *Listeria monocytogenes* toxin InlB contains a HGFR-interacting domain and is a functional analog of HGF. The aim of this work was to evaluate the hepatoprotective activity of the InlB HGFR-interacting domain. The recombinant HGFR-interacting domain InlB321/15 was purified from *E. coli*. MTT (3-(4,5-dimethylthiazol-2-yl)-2,5-diphenyltetrazolium bromide) test was used to measure InlB321/15 mitogenic activity in HepG2 cells. Activation of MAPK- and PI3K/Akt-pathways was tracked with fluorescent microscopy, Western blotting, and ELISA. To evaluate hepatoprotective activity, InlB321/15 and recombinant human HGF (rhHGF) were intravenously injected at the same concentration of 2 ng·g^−1^ to BALB/c mice 2 h before liver injury with CCl_4_. InlB321/15 caused dose-dependent activation of MAPK- and PI3K/Akt-pathways and correspondent mitogenic effects. Both InlB321/15 and rhHGF improved macroscopic liver parameters (liver mass was 1.51, 1.27 and 1.15 g for the vehicle, InlB321/15 and rhHGF, respectively, *p* < 0.05), reduced necrosis (24.0%, 16.18% and 21.66% of the total area for the vehicle, InlB321/15 and rhHGF, respectively, *p* < 0.05). Obtained data suggest that InlB321/15 is a promising candidate for a tissue repair agent.

## 1. Introduction

Proliferation of hepatocytes and liver stem cells underlies liver regeneration after acute toxic damage [1]. The HGF (hepatocyte growth factor)/HGFR (HGF receptor) signaling pathway is central to mitogenic, motogenic, and morphogenic effects in hepatocytes and hepatic progenitor cells [2,3]. Loss of the tyrosine kinase HGFR results in delay and abnormalities in the liver regeneration process [4]. Overexpression of HGF decreases acute liver injury and improves regeneration via activation of MAPK and PI3K/Akt/mTor signaling pathways and the antioxidant response [5,6]. However, the biological actions that are driven by the HGF/HGFR pathway all play roles in the progression of invasive and metastatic cancers [7,8]. Moreover, some pathogenic microorganisms hijack the host HGF/HGFR system to establish a comfortable environment for infection [8,9].

The InlB protein secreted by the Gram-positive pathogenic bacterium *Listeria monocytogenes* interacts with HGFR and causes HGFR autophosphorylation and downstream signaling cascade initiation [10,11]. InlB/HGFR interactions promote cell invasion, causing bacterial infection [9,11]. Purified InlB acts as a growth factor and stimulates cell proliferation and migration [11,12,13]. The InlB leucine-repeat rich (LRR)-domain flanked by specific *N*-cap and immunoglobulin-like domains, which are known together as an internalin domain or InlB321, is sufficient to activate HGFR controlled cascades [14,15].

Bacterial proteins have comparative advantages over recombinant human growth factors in regard to the accessibility, efficiency, and simplicity of production. Several studies have demonstrated the potential for InlB321 variants derived from different *L. monocytogenes* strains to stimulate the proliferation and motility of primary lung endothelial cells, keratinocytes, and Human Umblical Vein Endothelial Cells [13,14,15,16,17]. Recently, we showed that the variant InlB321 from *L. monocytogenes* strain VIMHA015 (InlB321/15) accelerated abrasion wound healing in mice [16]. Here, we demonstrated that InlB321/15 activated the HGFR-dependent MAPK and PI3K/Akt transduction pathways in HepG2 cells and exhibited hepatoprotective activity in the CCl4 induced liver injury mouse model.

## 2. Experimental Section

### 2.1. Animals

Female BALB/c mice (21–23 g) from the nursery Stolbovaya (Moscow region, Russia) were used throughout the experiments. Animals were maintained at a temperature of 20 ± 1 °C and a humidity of 50% and were subjected to a 12 h light/dark cycle with free access to food and water. Experiments on animals were conducted in accordance with the Russian Federation National Standard (GOST R52379-2005), directives of Ministry of Health of Russian Federation (No753n from 26.08.2010, No774н from 31.08.2010), and with the approval of the Biomedical Ethics Committee of Gamaleya Research Center of Epidemiology and Microbiology (No93 from 22.10.2015, approval date: 24 January 2017).

### 2.2. Cell Cultures

Human liver carcinoma HepG2 cells were obtained from the pubic collection of Gamaleya Center (Moscow, Russia). Cells were grown in the DMEM medium supplemented with 10% FBS (fetal bovine serum) in 5% CO_2_ atmosphere.

### 2.3. Antibodies and Growth Factor

Phospho-Erk1/2-specific, and HRP- or Alexa Fluor^®^ 488-conjugated anti-rabbit secondary antibodies were purchased from Abcam (ab 138482, ab97085 and ab150077, respectively) (Cambridge, UK). α-Tubulin-specific polyclonal antibody PA5-22060 and 6×-His Tag-specific monoclonal antibody MA1-21315 antibody were from purchased Thermo Fisher Scientific (Walltham, MA, USA). Phospho-Erk1/2-, and phospho-AKT-specific antibodies used in ELISA were included in the InstantOne ELISATM kit (Invitrogen^TM^, ThermoFisher Scientific). Recombinant human HGF (rhHGF) was purchased from Protein synthesis (Moscow, Russia; PSG190-10/27F112F1) and used according to the manufacturer’s instructions.

### 2.4. InlB321/15 Purification

InlB321/15 was purified via His-tag with Dynabeads^TM^ (Invitrogen^TM^, Thermo Fisher Scientific) from the recombinant *E. coli* BL21::pET28b::inlBallele9 strain as described [13]. The pET26b::inlBallele9 plasmid carried the InlB internalin domain encoding part of inlB gene from the virulent *L. monocytogenes* strain VIMHA015 isolated from a stillborn baby with listeriosis in Russia [13]. Protein purity was confirmed with SDS-PAGE (data not shown). Purified protein was dissolved at a concentration of 1.16 mg·mL^−1^ and maintained at +4 °C until use.

### 2.5. In Vitro Cell Viability Assay

A standard MTT cell proliferation and viability assay was performed in 96-well plates with the MTT reagent (R&D Systems, Minneapolis, MN, USA in triplicate. Results were read with the iMax reader (BioRad, city, state, country).

### 2.6. Immunofluorescence Microscopy

HepG2 cells were fixed and permeabilized with 3.7% paraformaldehyde and 0.1% Triton X-100, respectively. Cells were blocked with 2% BSA (in PBS) before incubation with primary antibodies overnight at 4 °C, followed by secondary antibody for 1 h at room temperature under dark conditions. Hoescht 33342 (Invitrogen) was then added, and samples were incubated under dark conditions for 20 min more. Confocal microscopy images were taken with a Carl Zeiss Axiovert fluorescent microscope at magnification 100× (Jena, Germany). The antibodies used are described above.

### 2.7. SDS-PAGE and Immunoblotting

Cells were lysed with RIPA buffer (25 mM Tris, pH8, 150 mM NaCl, 0.1% SDS, 0.5% sodium deoxycholate, 1% Triton X-100 and cocktail protease inhibitors). 4× Laemmli buffer (0.125 M Tris HCl, pH 6.8, 4% SDS, 20% glycerol, 10% β-mercaptoethanol, 0.001% bromophenyl blue) was added (3:1) to lysates, samples were boiled for 5 min, separated by electrophoresis on 10% SDS-PAGE, and visualized with Coomassie Brilliant Blue R-250. For immunoblotting, separated proteins were transferred onto nitrocellulose membrane, incubated with 5% BSA in Tris-buffered saline containing 0.1% Tween 20 for 1 h, blotted with anti-phospho-Erk1/2 antibodies (1:1000) at 4 °C overnight, and subsequently labeled with horseradish peroxidase-conjugated antibody against rabbit IgG (1:100,000) for 1 h. The signals were detected with ECL Plus detection reagents (Thermo Fisher Scientific).

### 2.8. InlB321/15 Toxicity Assay

Decimal dilutions of InlB321/15 from 2 to 2000 ng·g^−1^ diluted in PBS were injected intravenously into the tail veins of mice. Three mice in the group were included. The control group obtained PBS only. The mice were sacrificed and their livers were taken for macroscopic and histological analysis 48 h after the last injection. To determine body mass, two groups of 5 animals obtained the minimal dose of 2 ng·g^−1^ or vehicle twice a week for 3 weeks. The mice were sacrificed on the 21st day, and their blood was taken for ALT/AST analysis and the livers were taken for macroscopic and histological analysis.

### 2.9. CCl_4_ Acute Toxicity Testing

Mice were randomly allocated into 5 groups (*n* = 6 in each group). In groups 1, 2, and 4, acute liver injury was induced by the intragastric administration of a single dose of CCl_4_ dissolved in olive oil (1:1; 4.5 μL of blend oil per g). Intragastric injection was performed in animals starved for 12 h and anesthetized by intraperitoneal injection of sodium pentobarbital (40 µg/g). A needle with a diameter of 0.6 mm and a length of 25 mm was used to deliver 100 µL of CCl_4_ blend oil. The tip of the needle was cut and polished to smoothness to avoid stomach injury. Group 2 and 3 mice received an intravenous dose of InlB321/15 (2 ng·g^−1^) 2 h before intragastric injection of CCl_4_ or the same volume of solvent (olive oil) by intragastric injection without liver injury, respectively. Group 4 received HGF (2 ng·g^−1^) intravenously 2 h before CCl_4_ injection. Group 5 intravenously obtained a vehicle without InlB321/15 and the volume of solvent (olive oil) by intragastric injection. At 48 h after the CCl4 injection, the mice were bloodless and sacrificed under anesthesia. Their livers were removed, weighed, and fixed for subsequent histological examination as described below.

### 2.10. Histological Examination of Liver Sections

For histopathology, tissue sections were analyzed by hematoxylin-eosin staining. The necrotic area was determined in the microscopic field of a 3.56 mm^2^ area/section in 5 tissue sections obtained from different animals (*n* = 5) in each group, using morphometric analysis with ImageJ software on hematoxylin-eosin sections (Moscow, Russia).

### 2.11. Biochemical Markers

Serum AST and ALT levels were detected with the automated analyzer Chem Well 2900(T) (Awareness Technology, Ltd, Palm City, FL, USA) and mouse-specific reagents (Spinreact, Girona, Spain).

### 2.12. Statistics

Data are expressed as the mean ± standard deviation (SD). Two-group comparisons were conducted using the Student’s *t*-test, and comparisons of the means of 3 or more groups were performed by ANOVA. A value of *p* < 0.05 was considered to indicate a statistically significant difference.

## 3. Results

### 3.1. In Vitro InlB321/15 Mitogenic Activity

**Figure 1:**
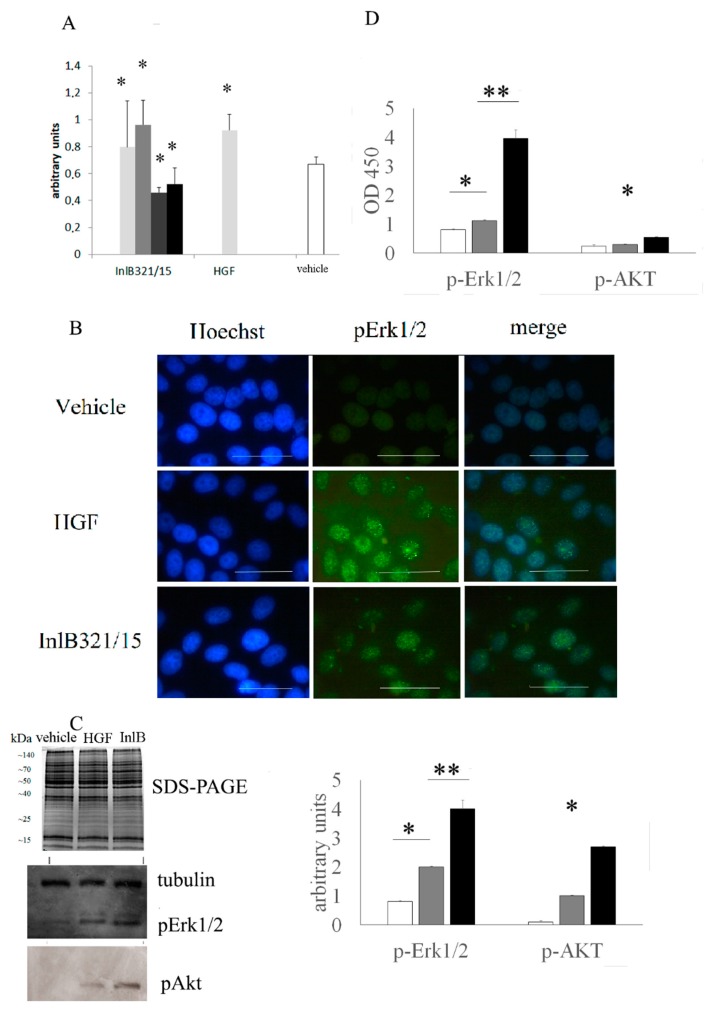
InlB321/15 activates Erk1/2 and Akt-controlled signaling pathways and provides a mitogenic effect in vitro. (**A**) MTT testing of HepG2 cell survival and proliferation. Cells were grown for 72 h with increasing InlB321/15 concentrations (100, 250, 500, or 1000 ng·mL^−1^) or rhHGF (100 ng·mL^−1^, as recommended by a manufacturer); (**B**) distribution of phospho-Erk1/2 in cells. Cells were treated with InlB321/15 or rhHGF for 15 min, then labelled with Hoechst to label nuclei, phospho-Erk1/2-specific antibodies and Alexa Fluor 488-labelled secondary antibodies, and observed with fluorescent microscopy; scale bar 50 µm; (**C**) Coomassie R-250 stained SDS-PAGE and Western blotting with anti-phospho-Erk1/2, anti-phospho-Akt and anti-tubulin specific antibodies, cell lysates were obtained from cells treated with InlB321/15 (250 ng·mL^−1^) or rhHGF (100 ng·mL^−1^) for 15 min; the densitogram shows corresponding values (white, gray and black graphs for vehicle, HGF and InlB321/15, respectively); (**D**) phospho-Erk1/2 and phospho-Akt were measured with InstantOne ELISA^TM^ (Invitrogen) in cell lysates obtained as in (**C**), data designations as in (**C**). Data represents mean values ± SD; * *p* < 0.05, ** *p* < 0.01.

InlB321/15 proliferative potential was tested on HepG2 cells in the MTT assay (Figure 1A). InlB321/15 provided a dose-dependent effect on HepG2 cells 48 h post addition. InlB321/15 taken at 250 ng·mL^−1^ caused a 1.21-fold increase in cell counting compared to the control (*p* < 0.05). rhHGF taken at 100 ng·mL^−1^ (a concentration recommended by the manufacturer) caused a 1.37-fold increase (*p* < 0.05) at high concentrations of 500 and 1000 ng·mL^−1^, while InlB321/15 caused a decrease in viable cell counting, suggesting a mild cytotoxic effect.

### 3.2. In Vitro inlb321/15-Stimulated MAPK- and PI3K/Akt Signaling Pathway Activation

The MAPK and PI3K/Akt cascades are central to the HGFR controlled signaling. The extracellular-signal-regulated kinase 1/2 (Erk1/2) belongs to the MAPK family and controls the initial stages of the MAPK cascade, while Akt/PKB is a key kinase of the PI3K/Akt signaling pathway [18]. Cell immunostaining with anti-phospho-Erk1/2 antibodies showed that both rhHGF and InlB321/15 stimulated with Erk1/2 lead to phosphorylation and delivery of the phosphor-Erk1/2 to the nucleus which is a prerequisite for mitogenesis (Figure 1B). To compare the activities of InlB321/15 and rhHGF, Western blotting and ELISA were applied (Figure 1C,D). Western blotting demonstrated clear induction of Erk1/2 and Akt-controlled pathways in cells stimulated with 250 μg·mL^−1^ InlB321/15 or 100 μg·mL^−1^ rhHGF (Figure 1C). Semi-quantitative ELISA analysis showed at least 3.5- and 1.7-fold higher effects of InlB321/15 compared to rhHGF on Erk1/2 and Akt phosphorylation (Figure 1D; *p* < 0.01).

### 3.3. InlB321/15 Toxicity in Mice

**Figure 2:**
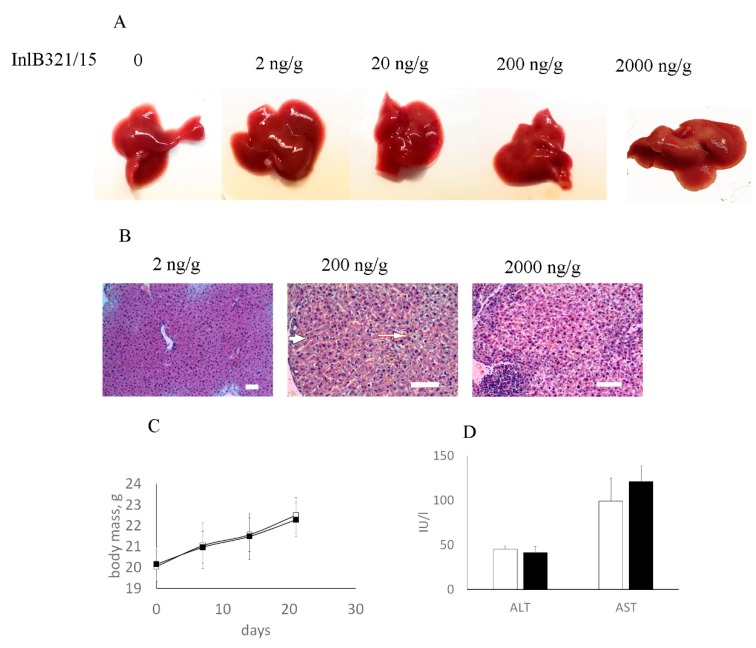
InlB321/15 toxicity assay in BALB/c mice. (**A**,**B**) Mice were injected with vehicle (PBS) or decimal dilutions of InlB321/15 (2–2000 ng per g of animal weight, g^−1^). The liver was taken for macroscopic (**A**) and pathohistological (**B**) examination at 48 h post treatment. The arrow shows dystrophic changes in the livers of animals that were given 200 and 2000 ng·g^−1^ InlB321/15; scale bar 100 µm. (**C**,**D**) A quantity of 2 ng·g^−1^ InlB321/15 was injected twice a week for 3 weeks; body masses were measured weekly (**C**); at the end of experiment, serum was taken to measure the injury markers amino transferase (ALT) and aspartate amino transferase (AST) (**D**).

Toxic in vitro effects of InlB321/15 when it was taken in high doses suggested potential toxicity in vivo. To check its toxicity for mice, InlB321/15 taken at concentrations from 2 ng per g of animal weight (g^−1^) to 2000 ng·g^−1^ was injected intravenously, and the liver was taken for macroscopic and histological analysis at 48 h post injection. No macroscopic changes were observed with the lowest dose of 2 ng·g^−1^, while higher doses caused light changes in the liver color (Figure 2A). Histological analysis revealed changes in liver tissue at higher doses. Hepatocyte dystrophy was observed after application of 200 ng·mL^−1^ InlB321/15 and dystrophic changes increased at a concentration of 2000 ng·g^−1^ (Figure 2B). Meanwhile, the liver tissue structure was totally unimpaired at a concentration of 2 ng·g^−1^, and it was therefore considered a no-observed-adverse-effect concentration. Weekly monitoring showed similar dynamics in body mass for animals which received proteins in the concentration 2 ng·g^−1^ and vehicle two times per week (Figure 2C). InlB321/15 injections did not change biochemical parameters characterizing cell damage, such as the presence of alanine amino transferase (ALT) and aspartate amino transferase (AST) in serum, as measured on the 21st day of the experiment (Figure 2D). Histological examination of the liver taken on the 21st day did not reveal pathological changes associated with InlB321/15 applications at this concentration.

### 3.4. InlB321/15 Hepatoprotective Activity

**Figure 3:**
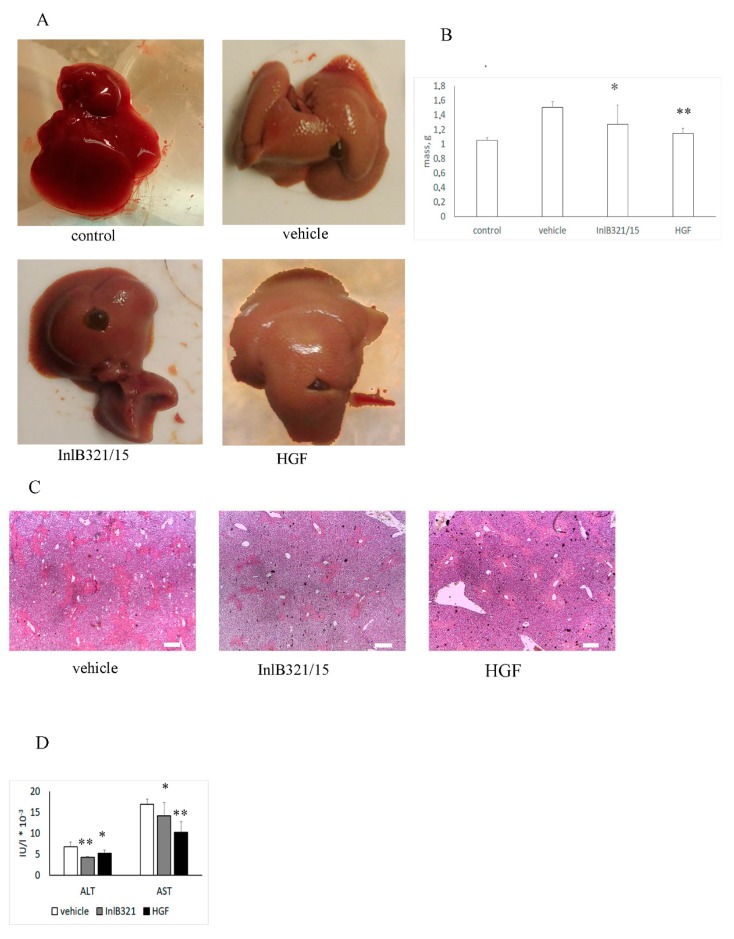
InlB321/15 provides a hepatoprotective effect. Mice received CCl4 intragastrically. A dose of 2 ng·g^−1^ InlB321/15 or 2 ng·g^−1^ rhHGF was injected intravenously 2 h before liver injury. The liver was extracted 48 h post liver injury. (**A**) Macroscopic changes in the liver; (**B**) changes in liver mass; (**C**) histopathologic changes in the liver; scale bar 250 µm; (**D**) amino transferase (ALT) and aspartate amino transferase (AST) accumulation in the serum. Data represent mean values ± SD; * *p* < 0.05, ** *p* < 0.01.

Obtained results demonstrated that 2 ng per gram of animal weight (g^−1^) InlB321/15 is safe for mice. We used this concentration to check the hepatoprotective effect of InlB321/15 for acute liver injury. rhHGF was taken in the same concentration as a control. InlB321/15 in a concentration of 2 ng·g^−1^ or 2 ng·g^−1^ rhHGF was injected intravenously 2 h before liver injury caused by intragastric injection of CCl_4_ (see Section 2). The livers were collected 48 h post injury. Visual comparison of livers demonstrated that InlB321/15 reduced the macroscopic destruction characteristics of acute CCl_4_ liver injury (Figure 3A). The visual data were supported by a relative decrease in liver mass compared with animals that obtained a vehicle (Figure 3B). The average liver masses were 1.51, 1.27, and 1.15 g for animals that obtained a vehicle, InlB321/15, and HGF, respectively. Histological analysis revealed multiple foci of centrilobular, periportal, and focal necrosis in CCl_4_-treated animals (Figure 3C). The application of InlB321/15 noticeably reduced the total area of necrosis. The necrotic areas were 24.0%, 16.18%, and 21.66% of the total area for animals that obtained a vehicle, InlB321/15 and rhHGF, respectively. Focal necrosis was almost absent when InlB321/15 was preventively applied. Application rhHGF noticeably reduced focal necrosis, too. Both InlB321/15 and rhHGF decreased ALT and AST concentrations in the serum (Figure 3D).

## 4. Discussion

Here, we demonstrated that the InlB321/15 protein, which is a HGFR ligand derived from the clinical *L. monocytogenes* strain, is a potential inducer of both MAPK and PI3K/Akt pathways. In accordance with these activities, InlB321/15 produced a mitogenic effect in HepG2 cells. The MAPK and PI3K/Akt pathways are central to HGF/HGFR-dependent tissue regeneration. We demonstrated that the protein InlB321/15, which is of bacterial origin, provided a hepatoprotective effect in the mouse CCl4 acute liver injury model. Statistically significant reduction of acute injury markers in mouse serum and a decrease in necrotic changes in the liver were observed after application of 2 ng·g^−1^ InlB321/15 2 h before toxic injury.

The HGFR tyrosine kinase receptor, also known as c-Met, is of central importance during embryogenesis, whereas it seems not to be vitally important in adult organisms under physiological conditions [2,19]. Nonetheless, HGFR plays a key role in the adaptive response of the liver to injury [2,4,20]. The role of HGFR in regeneration is based on anti-apoptotic and proliferative transduction pathways that begin with HGFR/HGF signaling. Particularly, activation of the Erk1/2 kinase, which is central to the MAPK cascade, during liver regeneration depends on HGFR [20,21]. Activation of the PI3K (for phosphatidylinositol 3-kinase)/Akt pathway provides both anti-apoptotic and proliferative signals important for liver regeneration [22,23,24]. HGFR activation via delivery of recombinant HGF or HGF-expressing plasmids and stem cells decreases lethal liver failure, prevents hepatocyte apoptosis, protects hepatocytes against oxidative injury, and decreases liver fibrosis [2,3,5,25].

Despite its obvious potential therapeutic uses, recombinant human HGF is restricted by its short life-time time in the blood-stream requiring multiple injections for a pronounced effect [5,26]. The complexity of the HGF structure does not allow prokaryotic expression, making large-scale pharmaceutical manufacturing more complicated and more costly [27]. To overcome these problems, plasmid-driven HGF-based medications were developed. Phase I–II clinical trials demonstrated that intramuscular injection of a HGF-expressing plasmid is safe, and may provide symptomatic relief for patients with critical limb ischemia [28]. Still, applications of HGF-expressing plasmids might be restricted because of the known roles of HGF and HGFR in tumorigenesis [7]. Using functional HGF analogs is an alternative to HGF-based approaches in regenerative medicine. Recently, we demonstrated that the InlB321/15 protein, which is a HGFR ligand of bacterial origin, accelerates abrasion wound closure [16]. Results obtained in this work demonstrated that the InlB321/15 protein possesses hepatoprotective activity comparable with rhHGF taken in the same concentration. Whether InlB321/15 might have a tumorigenic effect has not been studied yet. It seems negligible for single applications. The potential hazards of InlB321/15 daily applications have to be tested. Meanwhile, InlB321/15 does not require post-translational modifications and can be expressed in prokaryotic expression systems, making its manufacturing cost-effective and easy to control.

## 5. Conclusions

Obtained results demonstrated that InlB321/15 possesses hepatoprotective activity, diminishing liver injury caused by intragastric application of CCl_4_ that is comparable to or exceeds the activity of recombinant HGFR ligand (rhHGF). On the whole, our data suggest that InlB321/15 is a promising candidate to be used as a parenchymal tissue repair agent.
